# Risk Prediction of Edentulism in Chinese Adults: Insights From the China Health and Retirement Longitudinal Study (CHARLS)

**DOI:** 10.1016/j.identj.2025.109352

**Published:** 2025-12-25

**Authors:** Li Wang, Junfeng Wu, Ben Li, Ziqi Yang, Yihao Pei, Xiping Chen, Kan Xu

**Affiliations:** a Department of Pediatric Dentistry, Dental Disease Prevention and Control Institute, Jiading, Shanghai, China; b Department of Stomatology, Anting Hospital, Jiading, Shanghai, China; c School of Medicine, Medical College of Jinzhou Medical University, Jinzhou, China; d School of Stomatology, Medical College of Jinzhou Medical University, Jinzhou, China; e Department of General Stomatology, Dental Disease Prevention and Control Institute, Jiading, Shanghai, China; f Department of Geriatrics, Zhongshan Hospital, Fudan University, Shanghai, China

**Keywords:** Edentulism, Risk prediction, C-reactive protein, Estimated glucose disposal rate, Decision curve analysis

## Abstract

**Background:**

Edentulism concentrates in older adults and is associated with poorer diet, elevated malnutrition risk, and adverse health trajectories. Interpretable, well-calibrated tools applicable in routine care are needed to identify those at risk and support timely, tooth-preserving management.

**Methods:**

We analyzed data from the nationally representative China Health and Retirement Longitudinal Study with baseline in 2011 to 2012 and follow-ups in 2013, 2015, and 2018. Adults with baseline dentition status and all prespecified predictors were eligible. Incident edentulism was ascertained at each wave. Predictors defined a priori included age, sex, body mass index, blood pressure, lipid profile, log-transformed C-reactive protein (ln-CRP), and estimated glucose disposal rate (eGDR). Four algorithms were trained in a development set and evaluated in an independent validation set: multivariable logistic regression, LASSO-penalized logistic regression, random forest, and extreme gradient boosting. Validation included area under the receiver-operating-characteristic curve (AUC) with nonparametric comparisons, calibration with logistic recalibration, decision-curve analysis across clinically relevant thresholds, and time-dependent AUC over follow-up.

**Results:**

Of 6130 adults, 5231 were dentate at baseline and formed the risk set; incident edentulism occurred in 2013 (n = 338), 2015 (n = 253), and 2018 (n = 308). Logistic regression yielded the highest discrimination (AUC 0.695) with stable performance across waves and over 2-7 years. Calibration closely matched the ideal after simple recalibration, and decision curves showed consistent net benefit versus treat-all and treat-none strategies. In the final model, age and C-reactive protein (ln-CRP) were dominant independent predictors; men had lower risk, and total cholesterol showed a modest inverse association. A nomogram was derived to enable point-of-care risk stratification.

**Conclusions:**

In a large, nationally representative cohort, an internally validated logistic regression model based on routinely available data showed moderate discrimination, good calibration, and potential clinical utility for predicting incident edentulism. Age and low-grade systemic inflammation were the main contributors to risk stratification and may help guide early, tooth-preserving care.

## Introduction

Population ageing, together with lifelong exposure to oral diseases, has concentrated the burden of edentulism in older adults and positioned it as a sustained public health and nutrition concern in both global and Chinese settings.^1^ Analyses from the Global Burden of Disease program indicate that the overall burden of oral disorders rose from 1990 to 2019 and clusters in advanced age, underscoring the challenge that impaired oral function poses to healthy longevity in ageing societies.^2^ Consistent with these global patterns, long-term national estimates from China show increasing prevalence and disability burden attributable to complete tooth loss, highlighting the need for early risk identification and intervention in local populations.^3^ From a nutritional perspective, longitudinal evidence links tooth loss to reduced dietary diversity, insufficient intake of energy, protein, and micronutrients, and progression of multimorbidity, suggesting that compromised oral function may influence long-term health trajectories via nutrition-related pathways.^4^ In Chinese older adults, fewer remaining natural teeth are associated with poorer dietary diversity and nutritional status, providing direct population-based support for risk assessment that integrates oral, nutritional, and systemic health domains.^5^

With respect to intervention pathways, contemporary clinical studies show that prosthodontic rehabilitation improves subjective masticatory ability and oral health–related quality of life, whereas improvements in objective nutritional biomarkers and diet quality are not uniformly consistent across trials.^6^ When prosthodontic rehabilitation is combined with individualized dietary counseling, randomized trials consistently show simultaneous improvements in nutritional biomarkers, dietary structure, and masticatory function, suggesting that integrating nutrition management with functional rehabilitation is more effective and practical than either intervention alone.^7^

At the level of measurable biology, low-grade systemic inflammation and metabolic susceptibility offer actionable bridging indicators that connect oral function, nutrition, and systemic health. We use log-transformed C-reactive protein (ln-CRP) to capture low-grade inflammation; a meta-analysis of randomized trials shows that periodontal therapy lowers circulating CRP, indicating that local inflammatory control can down-shift systemic inflammatory load, while nutrition-related clinical benefits are more convincingly observed when comprehensive, diet-inclusive strategies are implemented.^8^ For metabolism, the estimated glucose disposal rate (eGDR) is a practical surrogate of insulin sensitivity and is independently associated with atherosclerotic cardiovascular events in a large multiethnic cohort, complementing conventional metabolic indicators for system-level risk characterization.^9^ In general-population follow-up, eGDR shows a nonlinear association with all-cause and cardiovascular mortality, suggesting threshold effects that strengthen the rationale for its use in risk stratification.^10^

Despite accumulating epidemiologic evidence on edentulism, studies that develop interpretable, deployable prediction models and validate them in independent samples remain relatively scarce. Contemporary methodology recommends adherence to the TRIPOD framework and its updates, with evaluation that goes beyond discrimination to emphasize calibration and clinical net benefit to improve translational usefulness.^11^ Decision-curve analysis enables quantification of net benefit across clinically relevant thresholds and directly addresses whether a model does more good than harm in practice. In parallel, adequate calibration is widely regarded as a prerequisite for implementation and the Achilles’ heel of predictive analytics, warranting careful scrutiny and, when necessary, simple recalibration before external use.^12^

Against this backdrop, we leveraged the nationally representative, prospective China Health and Retirement Longitudinal Study (CHARLS) to predefine candidate predictors and to develop and independently validate a parsimonious, interpretable, and bedside-ready model for incident edentulism, with the aim of enabling early identification and risk-stratified intervention in routine care. Our evaluation plan reports both time-invariant and time-dependent discrimination, calibration, and clinical net benefit; time-dependent receiver operating characteristic (ROC) methodology was used to describe discrimination over follow-up, nonparametric methods were applied to compare correlated areas under the curve, and clinical usefulness was summarized by net benefit on decision curves.

## Materials and methods

### Study design and population

**Fig. 1:**
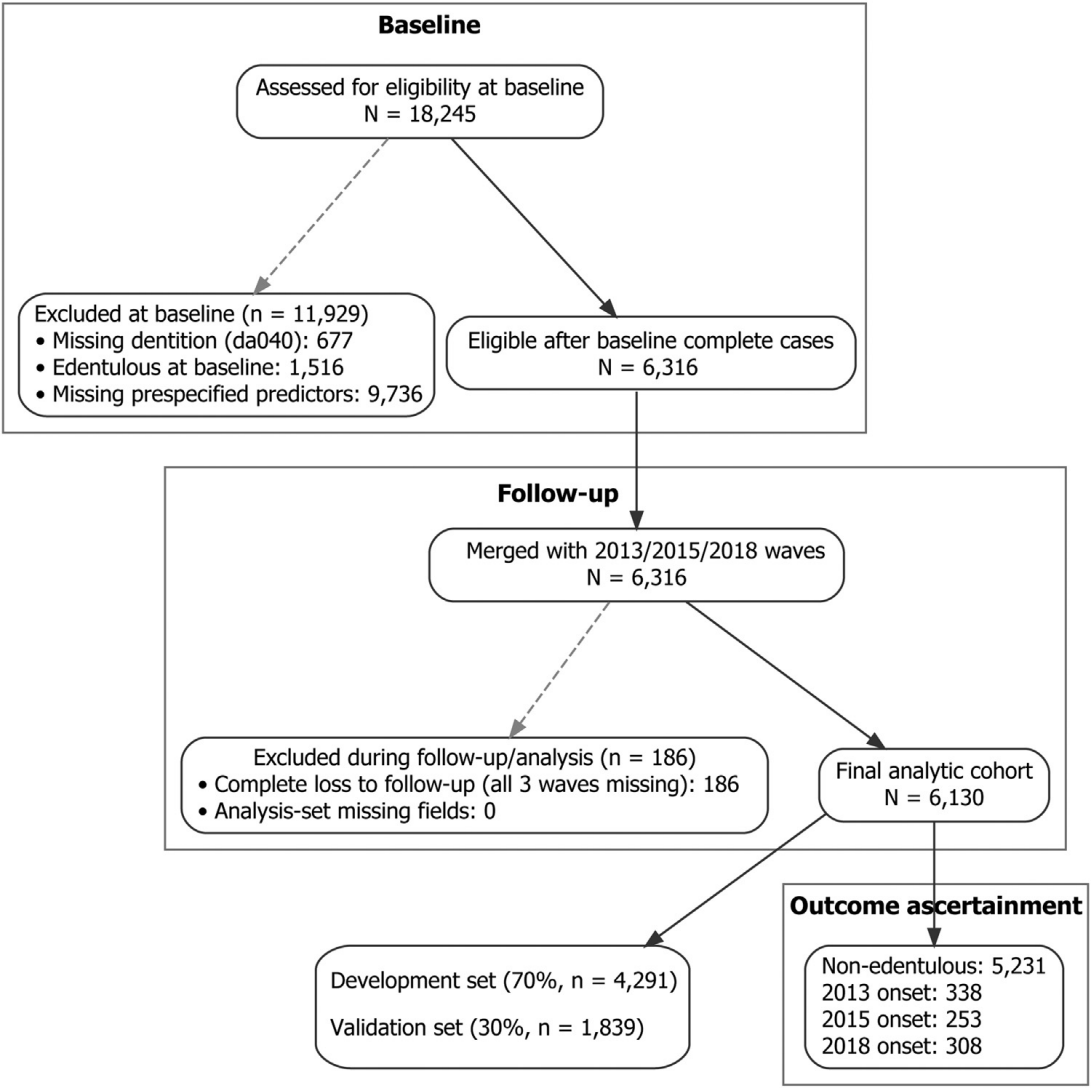
Participant flow from baseline to the final analytic cohort and outcome ascertainment in CHARLS, 2011-2018.

We used data from the China Health and Retirement Longitudinal Study (CHARLS) in a longitudinal cohort with a national baseline examination in 2011 to 2012 and follow-up assessments in 2013, 2015, and 2018, approximately 2, 4, and 7 years after baseline. Model development and validation were restricted to participants who were dentate at baseline; those already edentulous at baseline were summarized only in baseline descriptions. Participants were eligible if baseline dentition status and all prespecified predictors were recorded. Incident edentulism was ascertained at each follow-up according to standardized CHARLS procedures. Participant flow from baseline to the final analytic cohort is shown in Figure 1. In CHARLS, trained interviewers administered structured questionnaires that collected dentition status, sociodemographic characteristics and medical history, while anthropometric measurements, blood pressure and venous blood samples were obtained during a standardised health examination at the survey site.

### Eligibility and analytic samples

Eligibility required complete baseline dentition status and all candidate predictors. We prespecified 2 samples: a development set for model training and a distinct validation set defined before any model fitting. All multivariable analyses and figures used complete cases for the variables involved.

Of 18,245 participants assessed at baseline, 677 lacked baseline dentition status, 1516 were already edentulous, and 9736 were missing one or more prespecified predictors, leaving 6316 dentate complete cases eligible for linkage to follow-up waves. We then excluded 186 individuals with complete loss to follow-up, defined as dentition status missing at all 3 follow-up waves in 2013, 2015, and 2018. The final analytic cohort included 6130 participants. The development and validation sets were created by a 70:30 stratified random split by outcome, defined before model fitting to preserve event proportions. To characterise any selection introduced by the complete-case requirement for predictors, we additionally compared baseline characteristics of dentate participants who were retained in the complete-case cohort (n = 6316) with those who were dentate at baseline but excluded because of missing prespecified predictors (n = 9736). These descriptive comparisons are reported in Supplementary Table S1.

### Outcome ascertainment and follow-up handling

Dentition status came from CHARLS variable da040, coded 1 for edentulous and 2 for dentate. Incident edentulism was defined as the first follow-up wave with da040 = 1, corresponding to about 2, 4, or 7 years after baseline. Participants who never developed edentulism were censored at 7 years. Complete loss to follow-up was defined a priori as da040 missing at all 3 follow-up waves and led to exclusion. Within otherwise observed participants, wave-specific missing values were coded as no edentulism for that wave to reflect the discrete ascertainment schedule. All predictors were measured at baseline, and primary analyses used complete cases without imputation.

### Predictors and outcome

Candidate predictors specified a priori included age, sex, body mass index, systolic and diastolic blood pressure, total cholesterol, high-density and low-density lipoprotein cholesterol, ln-CRP, and eGDR.^9^ Because ln-CRP was right-skewed, it was modelled on the natural-log scale. These variables were chosen before any modelling on the basis of clinical plausibility, routine availability in CHARLS health examinations, and contemporary methodological guidance on prediction model development, which emphasises prespecification of a limited set of clinically relevant, routinely collected predictors and cautions against purely data-driven variable selection.^13^^,^^14^ Epidemiological and mechanistic studies also show that severe tooth loss and edentulism tend to co-occur with cardiometabolic conditions and low-grade systemic inflammation, so age, adiposity, blood pressure, lipids, ln-CRP and insulin-sensitivity surrogates such as eGDR together capture systemic pathways that link oral and general health. Although diabetes mellitus is an established risk factor for periodontal disease and tooth loss, we did not include fasting plasma glucose or diabetes classification as separate predictors. In CHARLS, fasting glucose was available only for a subset of participants and is more variable over the short term, whereas chronic glycaemic exposure is captured more stably by HbA1c, which is already incorporated into the eGDR formula. Self-reported diabetes status is also closely correlated with HbA1c and eGDR, so adding it as an extra predictor would mainly introduce redundancy in a model that we intentionally kept concise.

The eGDR (mg·kg⁻¹·min⁻¹) was calculated as:

eGDR=21.158−0.09×waistcircumference(cm)−3.407×hypertension−0.551×HbA1c(%),

with hypertension coded 1 for yes and 0 for no. The primary outcome was edentulism recorded at baseline and at each follow-up wave.

### Data handling

Continuous variables were screened for implausible values. Categorical predictors used conventional clinical reference levels. Unless stated otherwise, analyses were performed on complete cases and no imputation was used for the main results.

### Model development and validation

Four prespecified algorithms were trained in the development set and evaluated in an independent validation set: (1) multivariable logistic regression (LOGIT);^15^^,^^16^ (2) LASSO-penalized logistic regression with ten-fold cross-validation using the one-standard-error rule to select the penalty;^17^ (3) random forest;^18^^,^^19^ and (4) extreme gradient boosting.^20^ All models produced predicted probabilities on the 0 to 1 scale. Hyperparameters were specified a priori or chosen using standard defaults to limit overfitting; no posthoc tuning was based on validation performance.

We selected multivariable logistic regression as the primary modelling framework because the clinical aim was to estimate the probability of becoming edentulous over the approximate 7-year follow-up period, rather than the exact timing of edentulism. Follow-up in CHARLS occurs at 3 discrete survey waves, which produces interval censored event times, and logistic regression lends itself to a simple nomogram representation that can be readily applied at the point of care. In addition, before examining model performance we prespecified that, if logistic regression achieved discrimination and calibration that were similar to or better than those of the more complex algorithms, it would be prioritised for clinical translation on grounds of parsimony and interpretability.

### Performance assessment

Model discrimination in the validation set was summarised by ROC curves and the area under the curve (AUC), with nonparametric confidence intervals and standard errors obtained using established methods for correlated ROC curves.^21^^,^^22^ Overall accuracy was further assessed by the Brier score and by calibration intercepts and slopes. Calibration was examined by plots of observed versus predicted risk using LOESS smoothing; a logistic recalibration (Platt scaling) was fitted in the development set and applied to the validation set.^11^^,^^23^, ^24^, ^25^ Clinical usefulness was evaluated with decision-curve analysis over threshold probabilities of 0.05 to 0.40, comparing the model with “treat-all” and “treat-none” strategies.^23^^,^^26^^,^^27^ To provide threshold-specific classification metrics, we used the development set to identify the probability cut-off that maximised the Youden index and reported the corresponding sensitivity and specificity in the validation set. For descriptive risk stratification, predicted probabilities were computed for the full cohort, individuals were dichotomized at the cohort median, and survival separation was tested by the log-rank test. Variable-importance summaries were model-appropriate (absolute standardized coefficients for LOGIT; nonzero coefficients at the selected penalty for LASSO; impurity-based importance for random forest; split-gain importance for extreme gradient boosting) and were displayed using a harmonized lollipop layout for presentation.

### Discrimination over follow-up time

To characterize discrimination as a function of time since baseline, we estimated time-dependent AUCs under the cumulative–dynamic definition using inverse probability-of-censoring weights (IPCW) and derived nonparametric bootstrap confidence bands from 300 parallel resamples. Reporting times were prespecified at 2, 4, and 7 years. If a target time had too few risk sets or events for stable estimation, we reported the nearest evaluable time without imputation and noted this substitution in the figure and text.

### Subgroup analyses and baseline comparators

Prespecified subgroup performance was summarized by sex and by age dichotomized at the cohort median (57 years), reporting AUC (DeLong confidence intervals) and calibration slope (Wald intervals). Age was entered as a continuous predictor in all models, and the median-based age split was used only for descriptive assessment of performance consistency across age groups. For clinical benchmarking we compared LOGIT against 2 simple logistic models: age alone and age plus ln-CRP.

### Statistical analysis

Analyses were performed in R (version 4.5.1). Continuous variables are reported as mean (SD) and categorical variables as number (percentage). Group comparisons for baseline characteristics used one-way ANOVA for continuous variables and Pearson’s χ² tests for categorical variables. Two-sided *P* values <.05 were considered statistically significant unless otherwise specified. Given the predictive focus of the study and the prespecified set of candidate predictors, we did not apply formal adjustments for multiple comparisons; P values are presented as descriptive measures of the strength of evidence and are interpreted together with effect sizes and overall model performance.

In prespecified sensitivity analyses, we first refitted Cox proportional hazards models using the same set of predictors as in the multivariable logistic model, with time from baseline to incident edentulism or censoring as the outcome. These models provided adjusted hazard ratios and allowed us to examine whether effect directions were consistent when accounting for follow-up time. Second, we modelled eGDR flexibly using restricted cubic splines in the multivariable Cox model, with knots placed at the empirical quartiles and the median eGDR as the reference. Wald tests were used to assess the overall and nonlinear components of the spline term. Third, we generated sex-specific calibration curves in the validation set by plotting observed versus predicted risk with LOESS smoothing. These sensitivity analyses are presented in Supplementary Figures S2 to S4.

## Results

### Study population

Among 6130 adults, 5231 were dentate at baseline and contributed person-time at risk. Incident edentulism was ascertained at the 2013 (n = 338), 2015 (n = 253), and 2018 (n = 308) follow-ups. Compared with participants who remained nonedentulous, cases were older and had higher baseline CRP; educational attainment and marital status also differed across groups, whereas smoking and drinking did not (Table 1).

**Table 1:** Baseline characteristics of the study cohort by dentition status and onset year.

Characteristic	Overall (n = 6130)	Nonedentulous (n = 5231)	2013 onset (n = 338)	2015 onset (n = 253)	2018 onset (n = 308)	*P*-value
Gender n (%)						.622
Female	4087 (66.7)	3499 (66.9)	216 (63.9)	164 (64.8)	208 (67.5)	
Male	2043 (33.3)	1732 (33.1)	122 (36.1)	89 (35.2)	100 (32.5)	
Education n (%)						<.001
Below primary school	3034 (49.5)	2473 (47.3)	238 (70.4)	140 (55.3)	183 (59.4)	
Primary school	1343 (21.9)	1163 (22.2)	61 (18.0)	54 (21.3)	65 (21.1)	
Middle school	1204 (19.6)	1090 (20.8)	24 (7.1)	48 (19.0)	42 (13.6)	
High school or above	549 (9.0)	505 (9.7)	15 (4.4)	11 (4.3)	18 (5.8)	
Marital status n (%)						<.001
Married	5448 (88.9)	4705 (89.9)	275 (81.4)	219 (86.6)	249 (80.8)	
Other	682 (11.1)	526 (10.1)	63 (18.6)	34 (13.4)	59 (19.2)	
Smoke n (%)						.215
No	4425 (72.2)	3800 (72.6)	230 (68.0)	176 (69.6)	219 (71.1)	
Yes	1705 (27.8)	1431 (27.4)	108 (32.0)	77 (30.4)	89 (28.9)	
Drink n (%)						.525
No	4983 (81.3)	4252 (81.3)	276 (81.7)	212 (83.8)	243 (78.9)	
Yes	1147 (18.7)	979 (18.7)	62 (18.3)	41 (16.2)	65 (21.1)	
Hypertension n (%)						.034
No	4555 (74.3)	3917 (74.9)	248 (73.4)	171 (67.6)	219 (71.1)	
Yes	1575 (25.7)	1314 (25.1)	90 (26.6)	82 (32.4)	89 (28.9)	
Diabetes mellitus n (%)						.069
No	5745 (93.7)	4896 (93.6)	325 (96.2)	231 (91.3)	293 (95.1)	
Yes	385 (6.3)	335 (6.4)	13 (3.8)	22 (8.7)	15 (4.9)	
Heart problem n (%)						.023
No	5354 (87.3)	4588 (87.7)	299 (88.5)	211 (83.4)	256 (83.1)	
Yes	776 (12.7)	643 (12.3)	39 (11.5)	42 (16.6)	52 (16.9)	
Age mean (SD)	58.14 (9.21)	57.25 (8.93)	65.23 (9.59)	62.27 (8.83)	62.07 (8.50)	<.001
WC mean (SD)	84.69 (12.46)	84.92 (12.43)	82.96 (11.41)	84.42 (13.70)	83.02 (12.82)	.003
SBP mean (SD)	129.95 (21.74)	129.66 (21.30)	131.75 (22.02)	130.95 (22.18)	132.18 (27.56)	.071
DBP mean (SD)	75.64 (11.95)	75.88 (11.98)	74.00 (11.92)	74.55 (11.94)	74.26 (11.42)	.002
TC mean (SD)	193.12 (38.14)	193.44 (38.03)	189.48 (36.66)	190.54 (36.55)	193.69 (42.58)	.198
TG mean (SD)	133.23 (91.82)	134.43 (93.37)	121.84 (74.25)	126.87 (79.96)	130.56 (91.07)	.056
BMI mean (SD)	326.31 (8461.16)	304.04 (8331.60)	22.31 (3.82)	827.47 (12787.93)	626.42 (10586.11)	.627
HDL-C mean (SD)	49.79 (13.97)	49.65 (13.85)	50.32 (14.27)	50.80 (15.72)	50.74 (14.13)	.291
LDL-C mean (SD)	117.28 (34.82)	117.46 (35.01)	114.70 (32.67)	115.60 (32.54)	118.45 (35.68)	.402
BUN mean (SD)	15.46 (4.45)	15.41 (4.44)	15.86 (4.58)	15.80 (4.42)	15.69 (4.42)	.123
CRP mean (SD)	2.51 (6.62)	2.42 (6.35)	2.72 (5.66)	3.58 (11.02)	3.02 (7.19)	.021
HbA1c mean (SD)	5.29 (0.82)	5.29 (0.81)	5.24 (0.87)	5.29 (0.86)	5.33 (0.82)	.563
eGDR mean (SD)	9.75 (2.15)	9.75 (2.13)	9.90 (2.10)	9.54 (2.32)	9.76 (2.33)	.269

Abbreviations: BMI, body mass index; BUN, blood urea nitrogen; CRP, C-reactive protein; eGDR, estimated glucose disposal rate; SBP/DBP, systolic/diastolic blood pressure; TC/HDL-C/LDL-C, total/high-/low-density lipoprotein cholesterol; WC, waist circumference.

Values are presented as mean (SD) for continuous variables and as n (%) for categorical variables. *P*-values compare participants who remained nonedentulous during follow-up with those who developed incident edentulism in 2013, 2015, or 2018, using one-way ANOVA for continuous variables and Pearson’s *χ²* tests for categorical variables. Overall represents all participants in the analytic cohort (dentate at baseline with complete predictor data).

Among the 16,052 adults who were dentate and had dentition status recorded at baseline, 6316 (39.3%) had complete data on all prespecified predictors and were therefore eligible for linkage to follow-up, whereas 9736 (60.7%) were excluded because of missing predictors (Supplementary Table S1). Compared with excluded dentate participants, those retained in the complete-case cohort were more often female, had lower educational attainment, and were less likely to smoke, and they showed slightly higher prevalences of hypertension, diabetes, and heart disease. Differences in most continuous cardiometabolic markers were small in absolute magnitude despite statistical significance driven by the large sample size; in particular, mean CRP concentrations were very similar between groups (2.62 vs 2.78 mg/L).

### Comparative discrimination and clinical separation

**Fig. 2:**
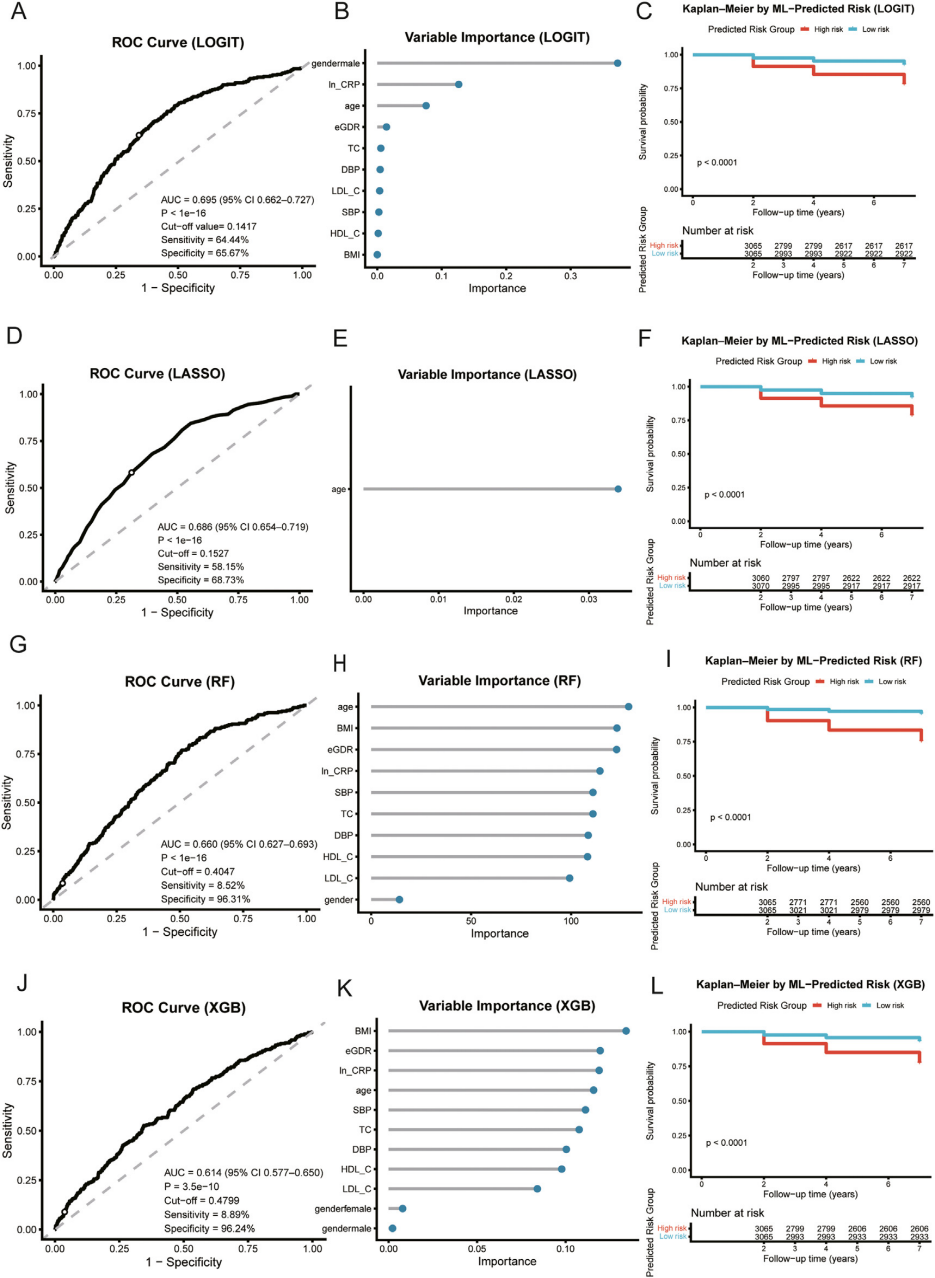
Comparative performance across 4 algorithms and clinical risk separation. A-C, logistic regression (LOGIT). D-F, LASSO. G-I, random forest. J-L, XGBoost. Left: ROC in the validation set (AUC shown). Middle: model-native variable importance in a harmonized lollipop layout. Right: Kaplan–Meier curves for the full cohort stratified by the model’s median predicted risk (log-rank *P*).

**Fig. 3:**
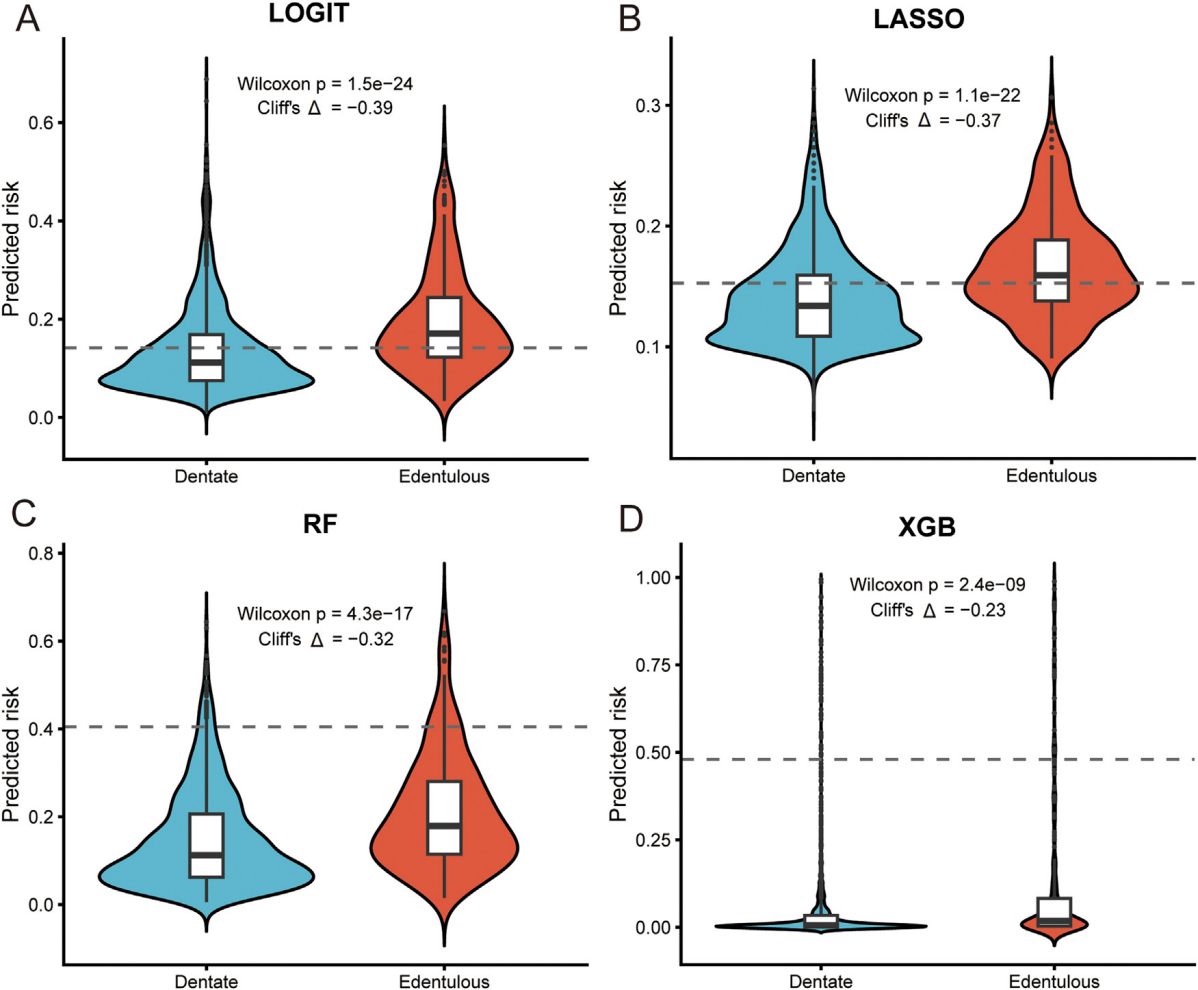
Predicted-risk distributions in the validation set. Violin and box plots for dentate versus edentulous outcomes for LOGIT A, LASSO B, random forest C, and XGBoost D,The dashed horizontal line marks the Youden cut-off estimated in the development set; panels report the Wilcoxon *P* value and Cliff’s *Δ.*

In the independent validation set, multivariable logistic regression (LOGIT) achieved the highest discrimination (AUC 0.695), followed by LASSO (0.686), random forest (0.660), and XGBoost (0.606) (Figure 2, left panels). Model-native importance consistently highlighted age and ln-CRP as the dominant signals, with male sex contributing toward lower predicted risk (Figure 2, middle panels). Kaplan–Meier curves stratified by each model’s median predicted risk showed clear separation with significant log-rank tests, indicating clinically meaningful risk stratification (Figure 2, right panels). Predicted-risk distributions were shifted upward among participants who became edentulous across all algorithms (Figure 3). Although the absolute AUC values were in the moderate range, the more complex algorithms did not yield a material gain in discrimination, and LOGIT showed the most favourable calibration profile. In this context, we judged that the modest numerical advantage of LOGIT, combined with its simplicity, made it the most appropriate choice for clinical use. For the penalized model, cross-validated tuning favoured the one-standard-error rule; coefficient paths and the deviance curve supported a sparse, stable LASSO solution (Supplementary Figure S1).

### Validation performance of the prespecified LOGIT model

**Fig. 4:**
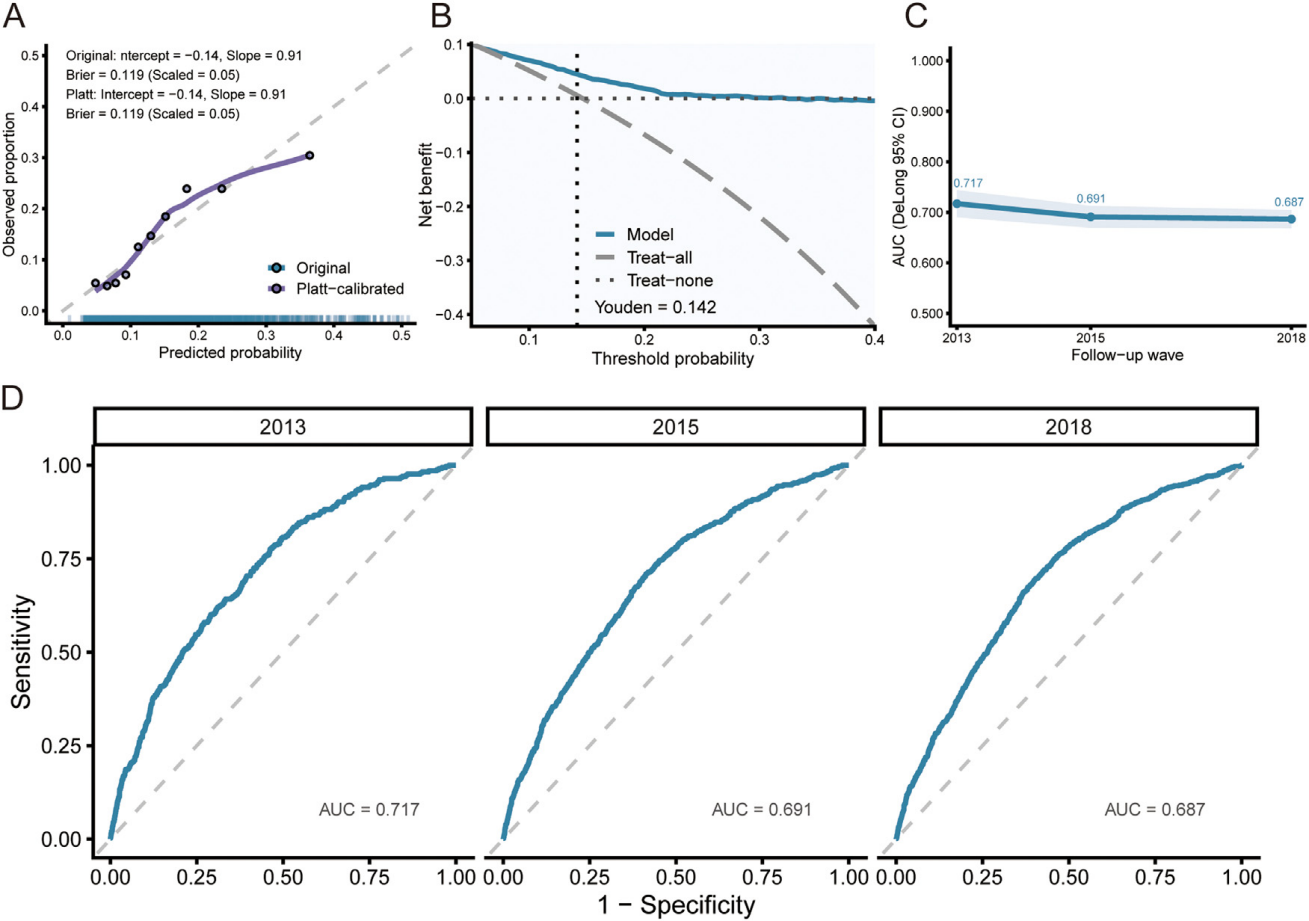
External performance of the prespecified LOGIT model. A, Calibration in the validation set with LOESS smoothing; Platt scaling was learned in the development set and applied to the validation set. B, Decision-curve analysis over threshold probabilities of .05 to .40. C, Time-slice validation by follow-up wave. D, Wave-specific ROC curves with AUCs.

Calibration in the validation set closely followed the 45° reference line, and Platt scaling learned in development produced only minor adjustment (Figure 4A). The overall Brier score was 0.119 (scaled 0.05), which is consistent with moderate accuracy for a population-based prediction tool. Decision-curve analysis indicated positive net benefit over threshold probabilities from 0.05 to 0.40 compared with “treat all” and “treat none” strategies (Figure 4B). Time-slice validation by follow-up wave yielded AUCs of 0.717 in 2013, 0.691 in 2015 and 0.687 in 2018 (Figure 4C-D). At the probability cut-off of 0.14, chosen by maximising the Youden index in the development set, sensitivity and specificity in the validation set were 64.4% and 65.7%, respectively.

### Discrimination over follow-up time

**Fig. 5:**
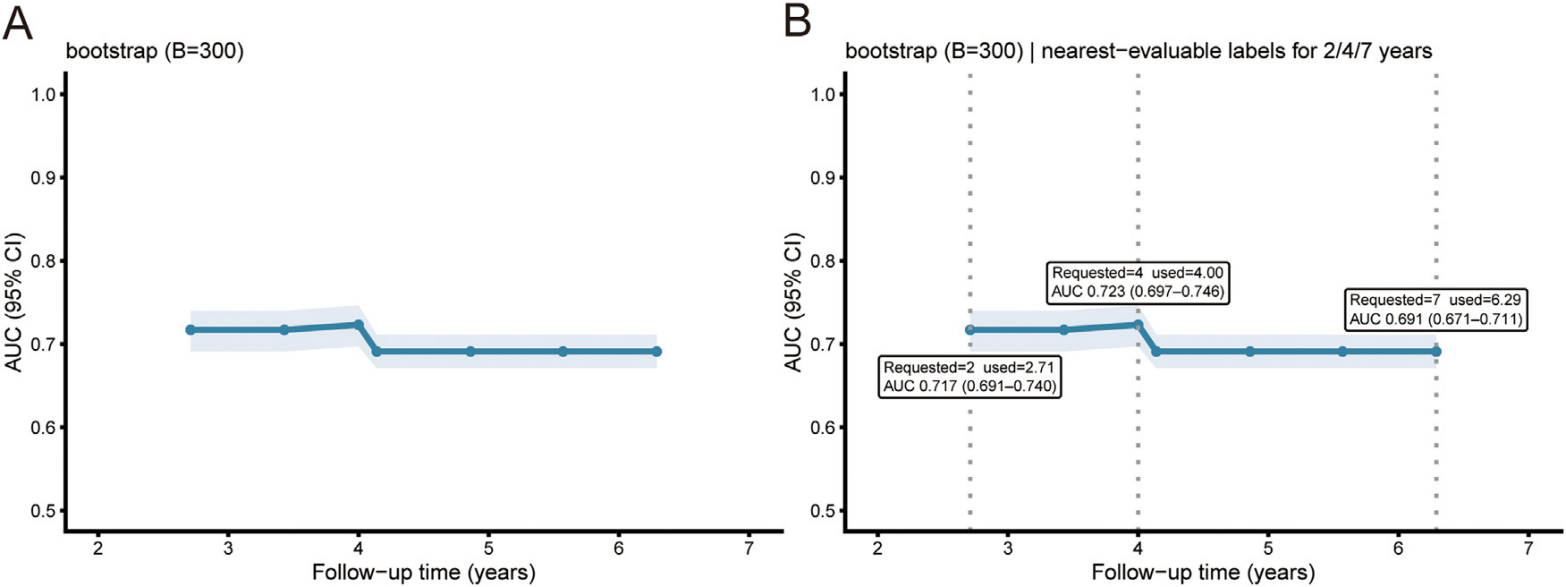
Time-dependent discrimination in the validation set (LOGIT). A, Time-dependent AUC with bootstrap 95% confidence band (300 resamples). B, Requested reporting times (2, 4, 7 years; grey dotted lines) and the nearest evaluable estimates used for computation (vertical marks), with corresponding AUCs shown in callouts.

Time-dependent AUC remained stable between 2 and 7 years (Figure 5). At prespecified reporting windows, the nearest evaluable estimates were 0.717 at 2.71 years (95% CI 0.691-0.740), 0.723 at 4.00 years (0.697-0.746), and 0.691 at 6.29 years (0.671-0.711) (Figure 5B). This stability of the time dependent AUC over 2-7 years suggests that the binary logistic framework does not mask major time related patterns in discrimination.

### Clinical decision benefit and subgroup validation

**Fig. 6:**
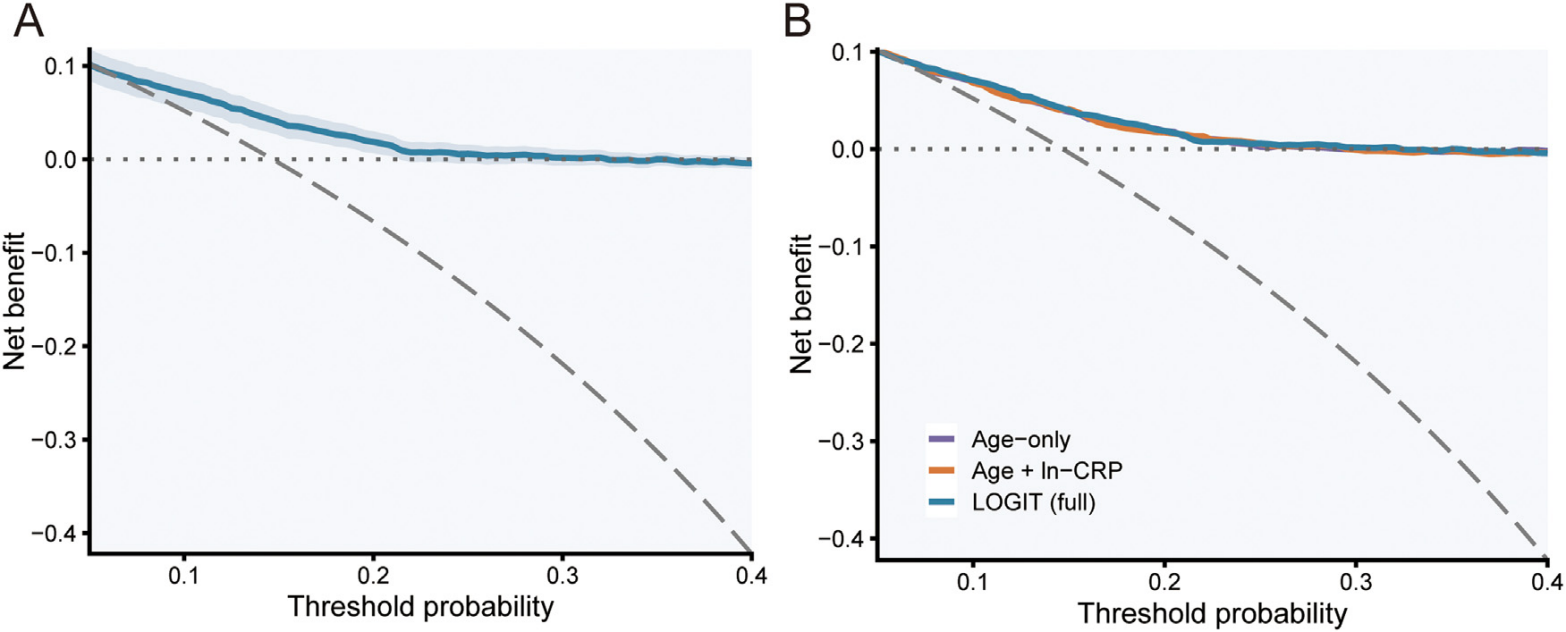
Decision-curve analysis in the validation set. A, LOGIT with bootstrap 95% CI (300 resamples). B, Benchmarking against clinical baselines (age-only; age plus ln-CRP) versus LOGIT. Grey long-dashed line denotes “treat-all”; dotted line denotes net benefit = 0.

**Fig. 7:**
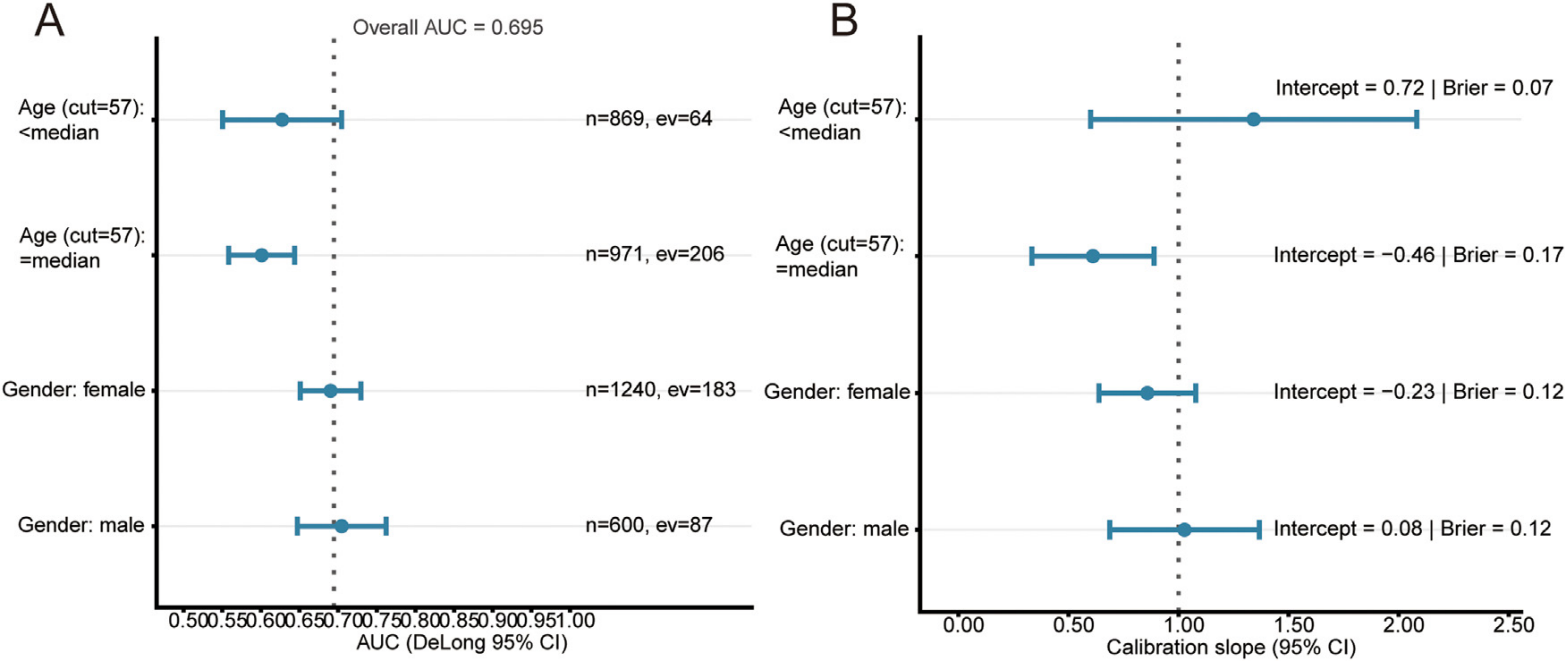
Subgroup validation for LOGIT in the independent test set. A, Forest plot of AUC (DeLong 95% CI) by sex and by age dichotomized at the cohort median (57 years); the vertical dotted line marks the overall AUC. B, Forest plot of calibration slope (Wald 95% CI) with a reference line at 1.

LOGIT showed net clinical benefit across a wide range of thresholds (Figure 6A). Against clinical baselines, LOGIT outperformed age alone and age plus ln-CRP and maintained comparable calibration (Figure 6B). Prespecified subgroup analyses by sex and by age dichotomized at the cohort median (57 years) yielded discrimination and calibration estimates broadly consistent with the overall validation set (Figure 7). Sex-specific calibration curves in the validation set further illustrated similar calibration patterns in females and males across the risk range (Supplementary Figure S2).

### Final model and nomogram

**Fig. 8:**
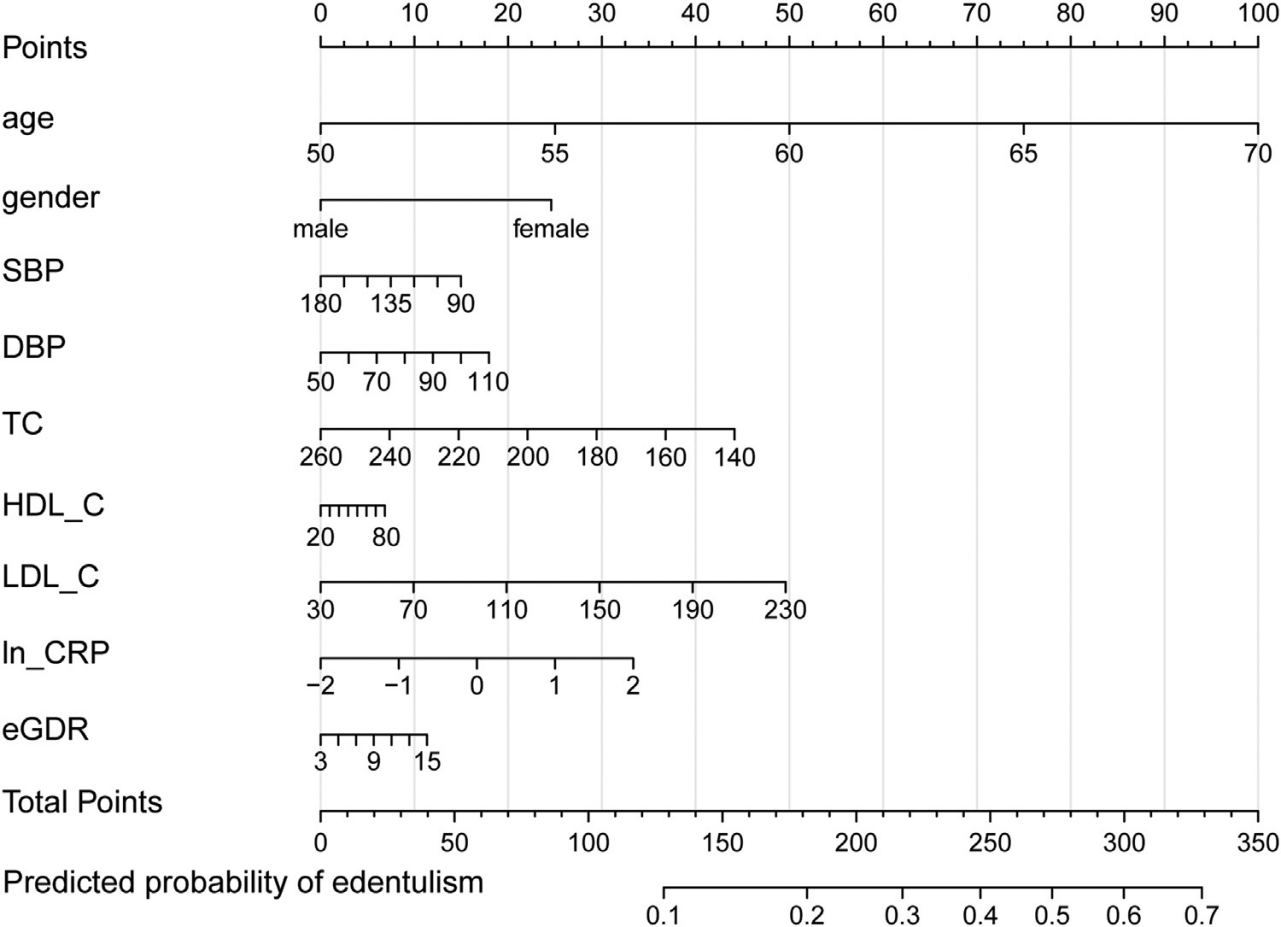
Nomogram for the final LOGIT model. Nomogram translating the multivariable logistic model into total points and the predicted probability of edentulism.

Given that multivariable logistic regression achieved the highest discrimination among the candidate algorithms, displayed favourable calibration, and is straightforward to interpret, we selected this model (LOGIT) for clinical presentation as a nomogram (Figure 8). In the adjusted model scaled per 10 units where applicable, older age was the dominant predictor (OR 2.13 per 10 years, 95% CI 1.91-2.38; *P* < .0001). Higher systemic inflammation was also associated with greater odds (ln-CRP: OR 1.13 per 1-log unit, 1.04-1.23; *P* = .003). Men had lower odds than women (OR 0.69, 0.57-0.84; *P* < .0001). Total cholesterol showed a small inverse association (OR 0.95 per 10 mg/dL, 0.90-1.00; *P* = .035). Other covariates (LDL-C, HDL-C, SBP, DBP, eGDR) had estimates near the null with confidence intervals crossing unity, indicating no statistically significant adjusted associations (Table 2).

**Table 2:** Multivariable logistic regression for predictors included in the nomogram.

Variable	β coefficient	Odds ratio (95% CI)	*P* value	Contrast
ln-CRP	0.13	1.13 (1.04-1.23)	.003	per 1 log unit
Gender (male vs ref)	−0.37	0.69 (0.57-0.84)	<.0001	vs reference
LDL-C	0.004	1.04 (0.98-1.10)	.18	per 10 mg/dL
DBP	0.005	1.05 (0.94-1.17)	.41	per 10 mmHg
TC	−0.006	0.95 (0.90-1.00)	.035	per 10 mg/dL
eGDR	0.014	1.01 (0.97-1.06)	.54	per 1 unit
SBP	−0.003	0.98 (0.92-1.04)	.42	per 10 mmHg
HDL-C	0.001	1.01 (0.95-1.09)	.68	per 10 mg/dL
Age	0.076	2.13 (1.91-2.38)	<.0001	per 10 years

Abbreviations: eGDR, estimated glucose disposal rate; HDL-C/LDL-C, high-/low-density lipoprotein cholesterol; ln-CRP, natural-log-transformed C-reactive protein; SBP/DBP, systolic/diastolic blood pressure; TC, total cholesterol.

ORs and 95% CIs are from the multivariable logistic regression model. For continuous predictors, effect scales follow the contrasts specified in the “Contrast” column. *P* values are from Wald tests.

### Sensitivity analyses

In sensitivity analyses, multivariable Cox proportional hazards models that used the same predictors as the logistic model showed a very similar pattern of associations (Supplementary Figure S3). Age remained positively associated with incident edentulism, male sex was protective, and higher education was associated with lower risk, whereas the estimates for lipids, blood pressure, BMI, BUN and eGDR clustered around the null. When eGDR was modelled as a restricted cubic spline in the adjusted Cox model, the association was nonlinear: risk was close to the null around the median but increased at both the lower and higher ends of eGDR, with statistically significant overall (*P* = .043) and nonlinear (*P* = .040) spline tests (Supplementary Figure S4). Sex specific calibration curves in the validation set showed broadly similar calibration for women and men across most of the predicted risk range, with some divergence only at the highest predicted probabilities where data were sparse (Supplementary Figure S2).

## Discussion

Leveraging the nationally representative, prospective CHARLS platform, we developed and independently validated prediction models for the incident endpoint of edentulism using prespecified predictors and algorithms, and confirmed that a multivariable logistic regression model delivered the most favourable balance of discrimination, calibration, and clinical usability. We further translated this model into a nomogram to support point-of-care communication and decision making.^28^ In the independent validation set, the model achieved an area under the ROC curve of 0.695, and wave-specific analyses yielded AUCs of 0.717 in 2013, 0.691 in 2015, and 0.687 in 2018, indicating stable medium-term discrimination. Uncertainty for comparisons of correlated AUCs was estimated using the DeLong method to ensure statistically coherent inference across models^29^. When discrimination was profiled over follow-up, the time-dependent AUC remained in the range of about 0.69 to 0.72 between 2 and 7 years, estimated under the established time-dependent ROC framework for censored outcomes proposed by Heagerty and colleagues, aligning the analysis with real-world longitudinal follow-up.^30^ Clinical usefulness, summarized by decision-curve analysis, showed positive net benefit across threshold probabilities from 0.05 to 0.40, consistently outperforming treat-all and treat-none strategies and thereby connecting model output to clinical trade-offs such as intervention burden and patient preferences.^31^^,^^32^ Transport performance in an independent hold-out sample from the same cohort received explicit attention: logistic recalibration learned in development required only minor adjustment when applied in validation, and calibration plots and slopes closely tracked the 45-degree reference, which is consistent with the principle that calibration is the Achilles’ heel of predictive analytics and a prerequisite for implementation^23^. On this basis, we present a nomogram as an interpretable, deployable interface; such individualized risk displays are already widely used in evidence-based oncology and surgery and offer a practical compromise between transparency and performance^33^. The main findings were robust in prespecified sensitivity analyses. Cox proportional hazards models that incorporated follow up time and sex specific calibration curves yielded effect estimates and calibration patterns that were closely aligned with those from the primary logistic framework, and flexible modelling of eGDR with restricted cubic splines confirmed the nonlinear association suggested by the main analyses (Supplementary Figures. S2-S4).

Although edentulism was ascertained at several follow-up waves, the primary clinical question in this study was whether an individual is likely to become edentulous over the medium term, approximately 7 years, rather than the exact timing of tooth loss. Logistic regression therefore provides a natural and transparent framework for modelling risk, particularly in the context of discrete survey waves and interval-censored event times, and its coefficients can be translated directly into a nomogram that is easy to implement in routine care. We complemented this approach with cox proportional hazards models and time-dependent ROC analyses, which produced very similar effect patterns and discrimination over follow-up, indicating that important time-related information was not lost when we summarised the endpoint as incident edentulism within 7 years. In interpreting performance we regarded the AUC as a summary of discrimination only and considered it together with calibration, Brier scores and decision-curve net benefit. A model with modest AUC may still be clinically useful if it is well calibrated and offers net benefit across the range of risk thresholds that are relevant for practice, which was the case here. At the cut-off that balanced sensitivity and specificity, both measures were around 65%, and the decision-curve analysis shows that clinicians can choose higher or lower thresholds to favour sensitivity or specificity depending on local priorities.

The dominant contribution of age observed here is consonant with 3 decades of global and Chinese evidence on oral-health burden, in which population ageing and lifelong oral-disease exposure concentrate severe tooth loss in older adults and reveal marked gradients by sex and socioeconomic position.^34^, ^35^, ^36^, ^37^ The positive association of baseline ln-CRP with incident edentulism aligns with the role of low-grade systemic inflammation in linking oral and systemic health. Population studies relate poorer periodontal health to higher CRP in both cross-sectional and longitudinal designs, and intensive periodontal therapy has been shown to lower systemic inflammatory markers, including interleukin-6 and CRP, indicating a modifiable connection between local and systemic inflammation.^34^^,^^38^, ^39^, ^40^, ^41^ Mechanistically, *Porphyromonas gingivalis* can induce pro-inflammatory cytokines that stimulate hepatic synthesis of CRP,^42^ and it can shift the RANKL–RANK–OPG axis toward bone resorption, promoting alveolar bone loss and functional decline.^43^, ^44^, ^45^ These pathways are congruent with immunosenescence under inflammaging, suggesting inflammation as a shared substrate for oral functional deterioration and multisystem risk in later life.^46^^,^^47^

From a metabolic perspective, eGDR serves as a surrogate of insulin sensitivity derived against the hyperinsulinemic–euglycemic clamp and has been validated in independent clamp datasets, supporting transportability across research settings.^48^ In large cohorts encompassing general and metabolically abnormal populations, lower eGDR is associated with higher risks of cardiovascular events and mortality, indicating epidemiologic relevance for capturing systemic metabolic–inflammatory susceptibility.^49^ In our restricted-cubic-spline sensitivity analysis, the association between eGDR and incident edentulism was nonlinear: risk approximated the null in the mid-range but rose at both low and high extremes, with statistically significant overall and nonlinearity tests. This pattern echoes recent reports of threshold or U-shaped associations between insulin-sensitivity surrogates and adverse outcomes. Such a pattern may reflect the combined influence of marked insulin resistance at the lower end of eGDR and body-composition variability or measurement heterogeneity among individuals with high eGDR values. Prospective validation in datasets that include dental specialty variables would help to disentangle these pathways.

Links between edentulism and nutrition continue to strengthen. Recent longitudinal evidence connects tooth loss to reduced dietary diversity, suboptimal protein and micronutrient intake, and adverse health trajectories, underscoring the role of nutritional pathways in functional decline.^4^ At the same time, prosthodontic rehabilitation alone does not consistently improve objective nutritional biomarkers, whereas combining rehabilitation with individualized dietary counseling yields more reproducible gains in nutritional markers, diet quality, and masticatory performance. These findings support a strategy of early identification and maintenance care, with nutrition interventions deployed when appropriate.^7^^,^^50^

For clinical translation, we recommend using the nomogram for individualized risk assessment in health checkups, chronic-disease follow-up, and integrated oral–primary care clinics, with threshold-based actions guided by the observed net-benefit range. As an operational example, we consider risk below 0.10 as low, warranting reinforced education and modestly increased follow-up frequency; risk from 0.10 to 0.20 as intermediate, warranting periodontal maintenance, early tooth-preserving rehabilitation, and concurrent management of inflammatory and metabolic burden; and risk at or above 0.20 as high, warranting specialist referral, intensified interventions, and individualized nutrition and metabolic assessment. Embedding the model in the electronic health record to auto-populate age, sex, CRP, and total cholesterol can enable real-time risk outputs and structured recommendations. Where target populations differ materially from our cohort, simple recalibration should precede deployment to preserve calibration and maintain clinical usefulness.

Several limitations merit consideration before implementation. First, the requirement for complete baseline biomarker data led to the exclusion of a substantial proportion of dentate participants. Among 16,052 adults who were dentate and had dentition status recorded at baseline, only 6316 (39%) had complete data on all prespecified predictors, whereas 9736 (61%) were excluded because of missing predictors (Supplementary Table S1). Compared with excluded dentate participants, those retained in the complete-case cohort were more often female, had lower educational attainment, were less likely to smoke and had slightly higher prevalences of hypertension, diabetes and heart disease, while mean CRP concentrations were similar. These patterns indicate that our analytic cohort best represents middle aged and older adults, especially women, with available biomarker measurements, and that selection bias and limited generalisability to other subgroups cannot be ruled out. Given that 61% of dentate adults with baseline dentition status were excluded because of missing predictors, inferences from the model should be interpreted as applying primarily to this selected subgroup rather than to the full CHARLS dentate population. Multiple imputation and weighting based sensitivity analyses in external data would help to assess the robustness of the model to such selection.

Second, edentulism, health behaviours and medical histories in CHARLS are obtained through interviewer administered questionnaires rather than clinical dental examinations or linkage to medical records. Complete tooth loss is usually salient for participants and the item explicitly refers to natural teeth, so gross misclassification of the outcome is probably uncommon, but some error is still possible, for example when participants confuse fixed prostheses or dentures with natural teeth. Self-report of hypertension and diabetes, which contribute to the eGDR calculation, and of smoking and drinking may also be imperfect. Such misclassification is expected to be largely non differential with respect to future edentulism and would tend to attenuate associations and reduce discrimination, so our estimates are likely to be conservative. Validation of questionnaire-based measures against clinical oral examinations and medical records would help to quantify this source of error. In addition, oral health information in CHARLS is limited to this binary item on complete tooth loss, without detailed tooth counts, periodontal indices or caries assessments, which restricts our ability to characterise gradients of oral disease severity and to distinguish different pathways leading to edentulism.

Third, incident events were ascertained at discrete waves, which limits temporal resolution; despite the use of time dependent AUC to better align with longitudinal risk, replication in datasets with finer event timing is warranted.

Fourth, residual confounding is possible because dental specialty exposures and behavioural factors were not included; future model updating should incorporate periodontal status, caries activity and oral hygiene behaviour and quantify added value using net reclassification improvement and net benefit metrics. Although we examined several predictors and subgroups, we did not apply formal adjustments for multiple comparisons, so some secondary associations may be due to chance and should be interpreted with caution.

Finally, fairness and access require proactive evaluation at deployment, with subgroup calibration and net benefit consistency checks across education level, urban and rural residence and smoking status to mitigate potential performance drift across populations. A key limitation of our work is that the model has so far been evaluated only within the CHARLS cohort, using an internal split-sample validation; its performance in other healthcare settings and populations remains uncertain. Future work should prioritize external validation across regions and care levels, followed by simple recalibration of intercept and slope and, when justified, limited parameter updating to preserve calibration transportability without sacrificing parsimony. In real-world deployment, action thresholds should be aligned with local resource constraints and patient preferences, using decision-curve analysis alongside cost–effectiveness evaluation to select thresholds and care pathways that yield net clinical benefit. As periodontal indices, caries activity measures, radiographic assessments, and social determinants become available, incremental value should be quantified with reclassification metrics and decision-analytic gain, while guarding against overfitting and maintaining interpretability and operational feasibility. Continuous monitoring of subgroup calibration and net-benefit consistency is also advisable to ensure equitable performance across educational strata, urban–rural settings, and smoking status once the model is embedded in routine care.

## Conclusion

Using data from the nationally representative CHARLS cohort, we developed and internally validated, in an independent hold-out sample, a parsimonious and interpretable logistic model for incident edentulism that demonstrated moderate discrimination, sound calibration, and clear clinical net benefit within this setting. Age and ln-CRP emerged as principal independent predictors, men had lower risk, and total cholesterol showed a modest inverse association. We provide a nomogram to facilitate risk stratification and early, actionable, tooth-preserving care across health checkups, primary care follow-up, and specialist collaboration, while recognising that external validation in other populations will be an essential next step before routine implementation.

## Institutional review board statement

This study used publicly available de-identified data from the China Health and Retirement Longitudinal Study (CHARLS). The CHARLS field surveys were approved by the Peking University Biomedical Ethics Review Committee (IRB00001052-11015; biomarker components, IRB00001052-11014), and written informed consent was obtained from all participants. The secondary analysis of the de-identified dataset was deemed exempt by the Institutional Review Board of the Jiading District Institute for Dental Disease Prevention and Control, Shanghai, China.

## Informed consent statement

Written informed consent was obtained by the CHARLS team from all participants. This study analysed publicly available de-identified CHARLS data and therefore no additional participant consent was required for the secondary analysis.

## Author contributions

**Li Wang:** Conceptualization, Methodology, Writing – original draft, Writing – review & editing, Visualization. **Junfeng Wu:** Conceptualization, Software, Validation, Writing – original draft, Visualization. **Ben Li:** Conceptualization, Methodology, Validation, Formal analysis, Writing – original draft, Writing – review & editing, Supervision. **Ziqi Yang:** Software, Validation, Writing – original draft, Writing – review & editing, Visualization. **Yihao Pei:** Methodology, Writing – original draft, Writing – review & editing. **Xiping Chen:** Formal analysis, Writing – review & editing, Supervision, Project administration. **Kan Xu:** Methodology, Software, Formal analysis, Writing – review & editing, Supervision, Project administration.

## Funding

This work was supported by the Basic Scientific Research Projects of the Liaoning Provincial Department of Education in 2023 (no. JYTMS20230677).

## Declaration of competing interest

None disclosed.

## Appendix Supplementary materials

Image, application 1

Image, application 2

Image, application 3

Image, application 4

Image, application 5

## Footnotes

Supplementary material associated with this article can be found in the online version at doi:10.1016/j.identj.2025.109352.
