# Supplementary material for: Risk Prediction of Edentulism in Chinese Adults: Insights From the China Health and Retirement Longitudinal Study (CHARLS)
Source: Int Dent J. 2025 Dec 25;76(1):109352. doi: 10.1016/j.identj.2025.109352 (PMC12800401; doi:10.1016/j.identj.2025.109352)
Supplement: Supplementary file 1 [file mmc1.docx]

Supplementary Materials:

Supplementary Table S1. Baseline characteristics of dentate participants included in the complete-case cohort versus those excluded because of missing prespecified predictors.

| Characteristic |  | Overall (n=16052) | Included (n=6316) | Excluded (n=9736) | p-value |
| --- | --- | --- | --- | --- | --- |
| age Mean (SD) |  | 58.02 (9.58) | 58.31 (9.31) | 57.84 (9.74) | 0.002 |
| WC Mean (SD) |  | 84.63 (17.00) | 84.66 (12.50) | 84.59 (20.58) | 0.831 |
| SBP Mean (SD) |  | 130.46 (24.29) | 130.05 (21.82) | 130.87 (26.57) | 0.059 |
| DBP Mean (SD) |  | 76.05 (12.19) | 75.62 (11.99) | 76.49 (12.39) | <0.001 |
| TC Mean (SD) |  | 192.94 (38.67) | 193.06 (38.14) | 192.77 (39.45) | 0.706 |
| TG Mean (SD) |  | 136.19 (112.91) | 132.82 (91.40) | 141.16 (138.46) | <0.001 |
| HDL_Mean (SD) |  | 50.68 (15.26) | 49.81 (14.00) | 51.96 (16.87) | <0.001 |
| LDL_ Mean (SD) |  | 115.88 (34.82) | 117.32 (34.81) | 113.76 (34.72) | <0.001 |
| BUN Mean (SD) |  | 15.71 (4.63) | 15.49 (4.56) | 16.04 (4.70) | <0.001 |
| CRP Mean (SD) |  | 2.69 (7.29) | 2.62 (7.06) | 2.78 (7.61) | 0.286 |
| Hba1c Mean (SD) |  | 5.26 (0.82) | 5.29 (0.83) | 5.23 (0.81) | <0.001 |
| eGDR Mean (SD) |  | 9.79 (2.14) | 9.74 (2.16) | 9.92 (2.10) | <0.001 |
| Gender |  |  |  |  | <0.001 |
|  | female | 8291 (51.7) | 4198 (66.5) | 4093 (42.1) |  |
|  | male | 7749 (48.3) | 2118 (33.5) | 5631 (57.9) |  |
| Education |  |  |  |  | <0.001 |
|  | below primary school | 6891 (42.9) | 3147 (49.8) | 3744 (38.5) |  |
|  | primary school | 3495 (21.8) | 1376 (21.8) | 2119 (21.8) |  |
|  | middle school | 3488 (21.7) | 1234 (19.5) | 2254 (23.2) |  |
|  | high school or above | 2178 (13.6) | 559 (8.9) | 1619 (16.6) |  |
| Marital status |  |  |  |  | 0.3 |
|  | married | 14262 (88.8) | 5591 (88.5) | 8671 (89.1) |  |
|  | other | 1790 (11.2) | 725 (11.5) | 1065 (10.9) |  |
| Smoking |  |  |  |  | <0.001 |
|  | No | 9744 (60.7) | 4539 (71.9) | 5205 (53.5) |  |
|  | Yes | 6300 (39.3) | 1777 (28.1) | 4523 (46.5) |  |
| Drinking |  |  |  |  | 0.666 |
|  | No | 9720 (81.0) | 5127 (81.2) | 4593 (80.8) |  |
|  | Yes | 2277 (19.0) | 1189 (18.8) | 1088 (19.2) |  |
| Hypertension |  |  |  |  | <0.001 |
|  | No | 12095 (75.8) | 4668 (73.9) | 7427 (77.1) |  |
|  | Yes | 3855 (24.2) | 1648 (26.1) | 2207 (22.9) |  |
| Diabetes |  |  |  |  | 0.006 |
|  | No | 14986 (94.3) | 5915 (93.7) | 9071 (94.7) |  |
|  | Yes | 908 (5.7) | 401 (6.3) | 507 (5.3) |  |
| Heart problem |  |  |  |  | <0.001 |
|  | No | 14089 (88.3) | 5509 (87.2) | 8580 (89.1) |  |
|  | Yes | 1859 (11.7) | 807 (12.8) | 1052 (10.9) |  |


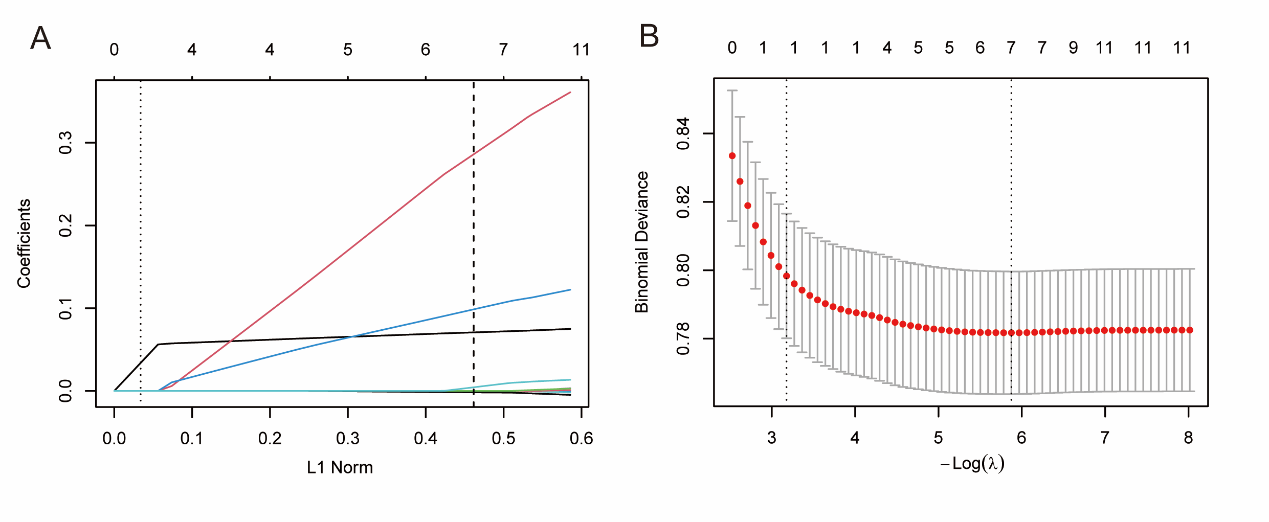


**Supplementary Figure S1.** LASSO model diagnostics.
(A) Coefficient paths as a function of the L1 norm; vertical lines mark λ_min and λ_1se.
(B) Tenfold cross-validated binomial deviance versus −log(λ) with ±1 SE bars. Primary analyses used λ_1se.


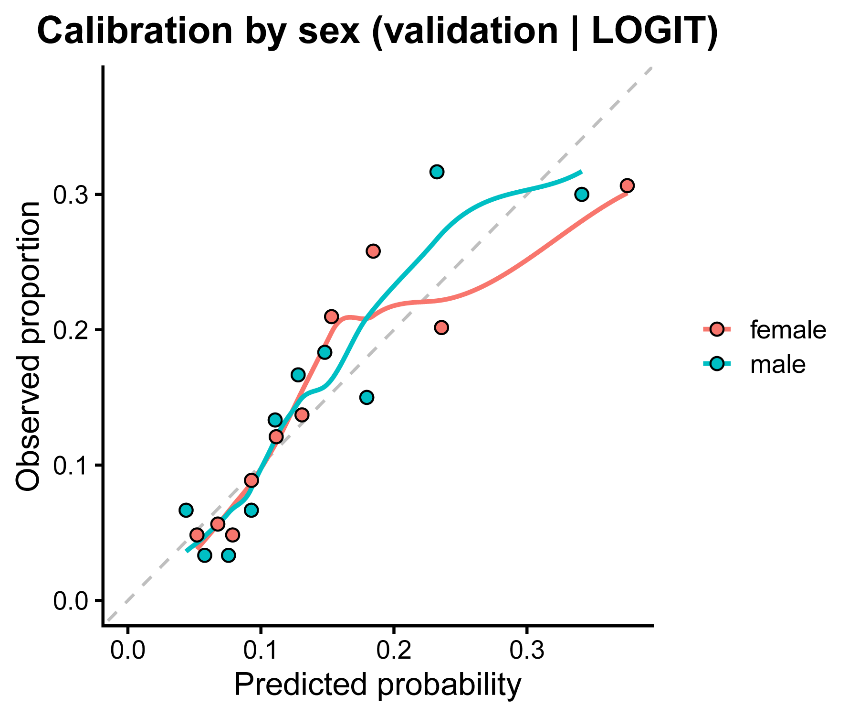


**Supplementary Figure S2.** Calibration by sex in the validation set (LOGIT).
LOESS-smoothed calibration curves for females and males; the grey dashed line indicates perfect calibration.


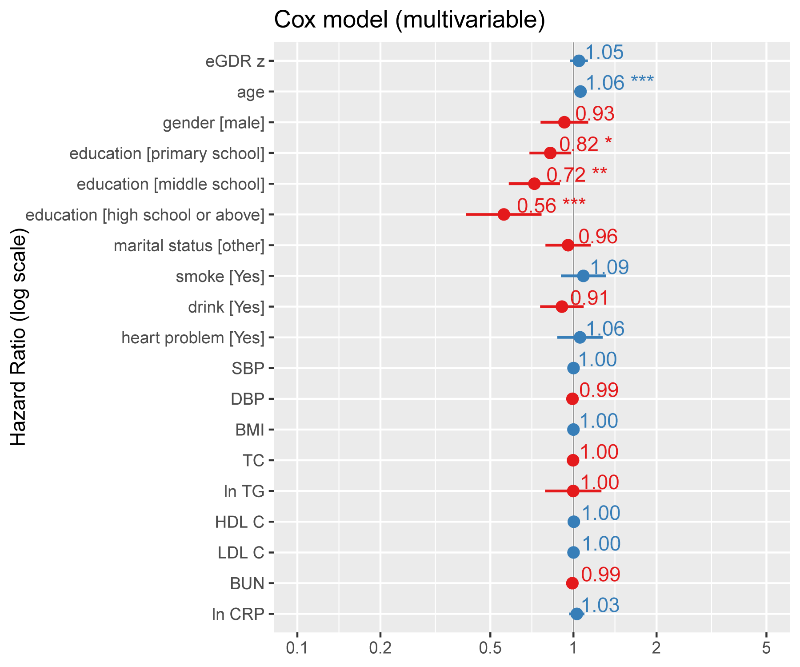


**Supplementary Figure S3.** Multivariable Cox model forest plot (adjusted hazard ratios).
Adjusted hazard ratios with 95% CIs on a log scale for the full covariate set.


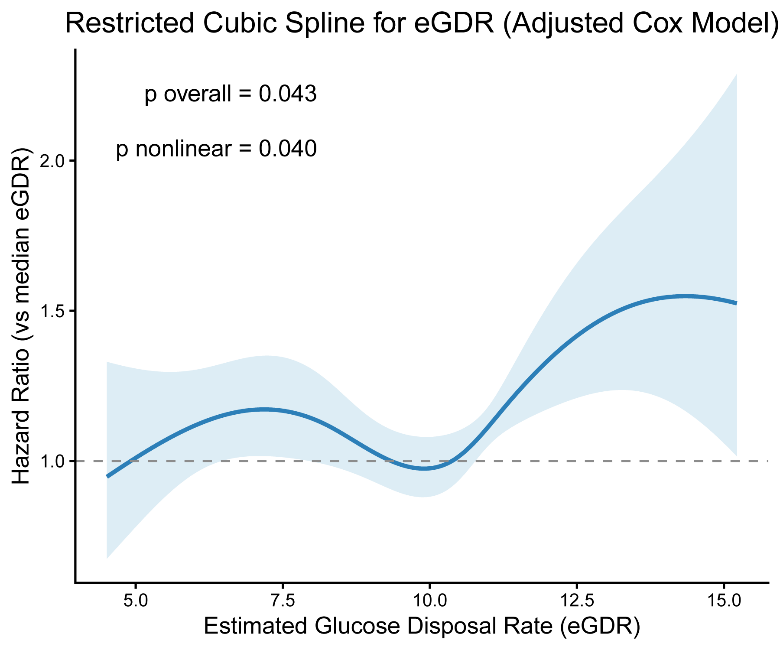


**Supplementary Figure S4.** Restricted cubic spline for eGDR in the Cox model.
Adjusted association between eGDR and edentulism risk modeled with restricted cubic splines.
