# Supplementary figures and images for: Risk Prediction of Edentulism in Chinese Adults: Insights From the China Health and Retirement Longitudinal Study (CHARLS)
Source: Int Dent J. 2025 Dec 25;76(1):109352. doi: 10.1016/j.identj.2025.109352 (PMC12800401; doi:10.1016/j.identj.2025.109352)

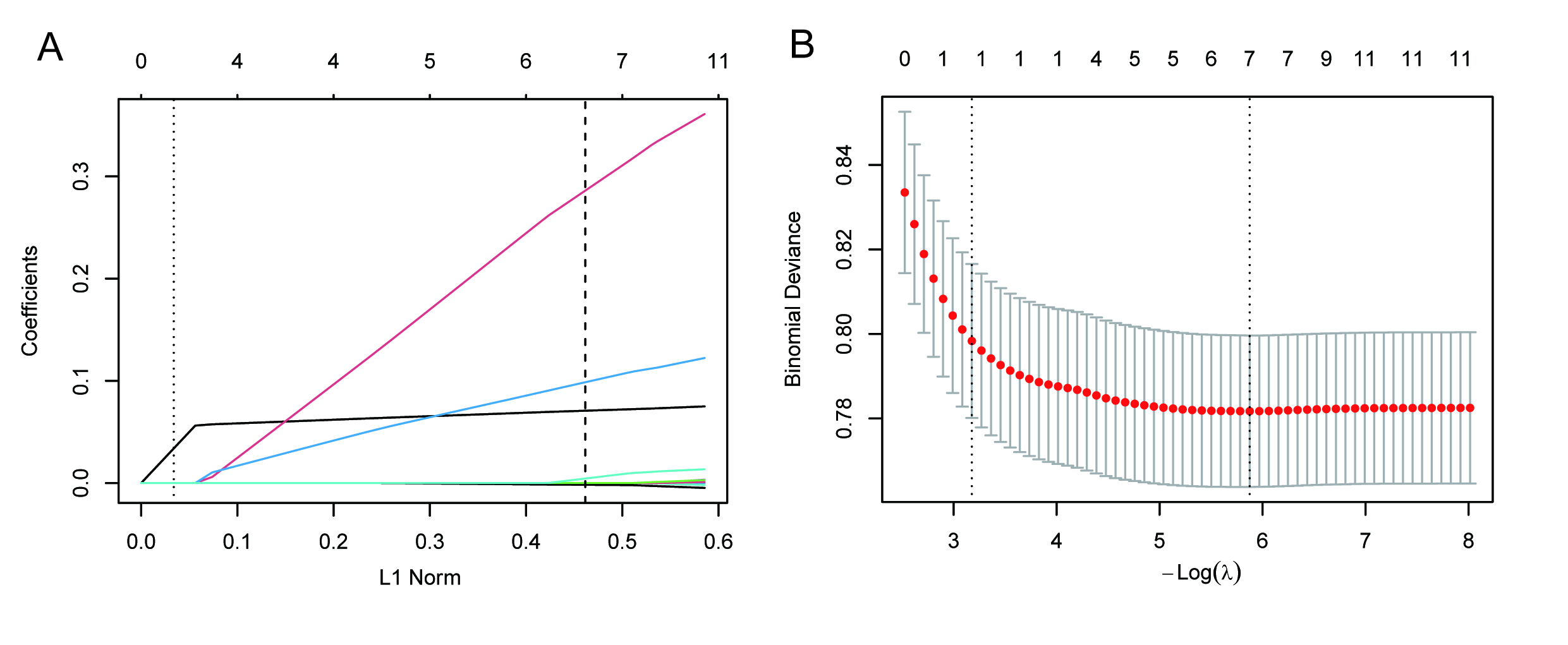

Supplement: Supplementary file 2 [file mmc2.zip › mmc2.tif]

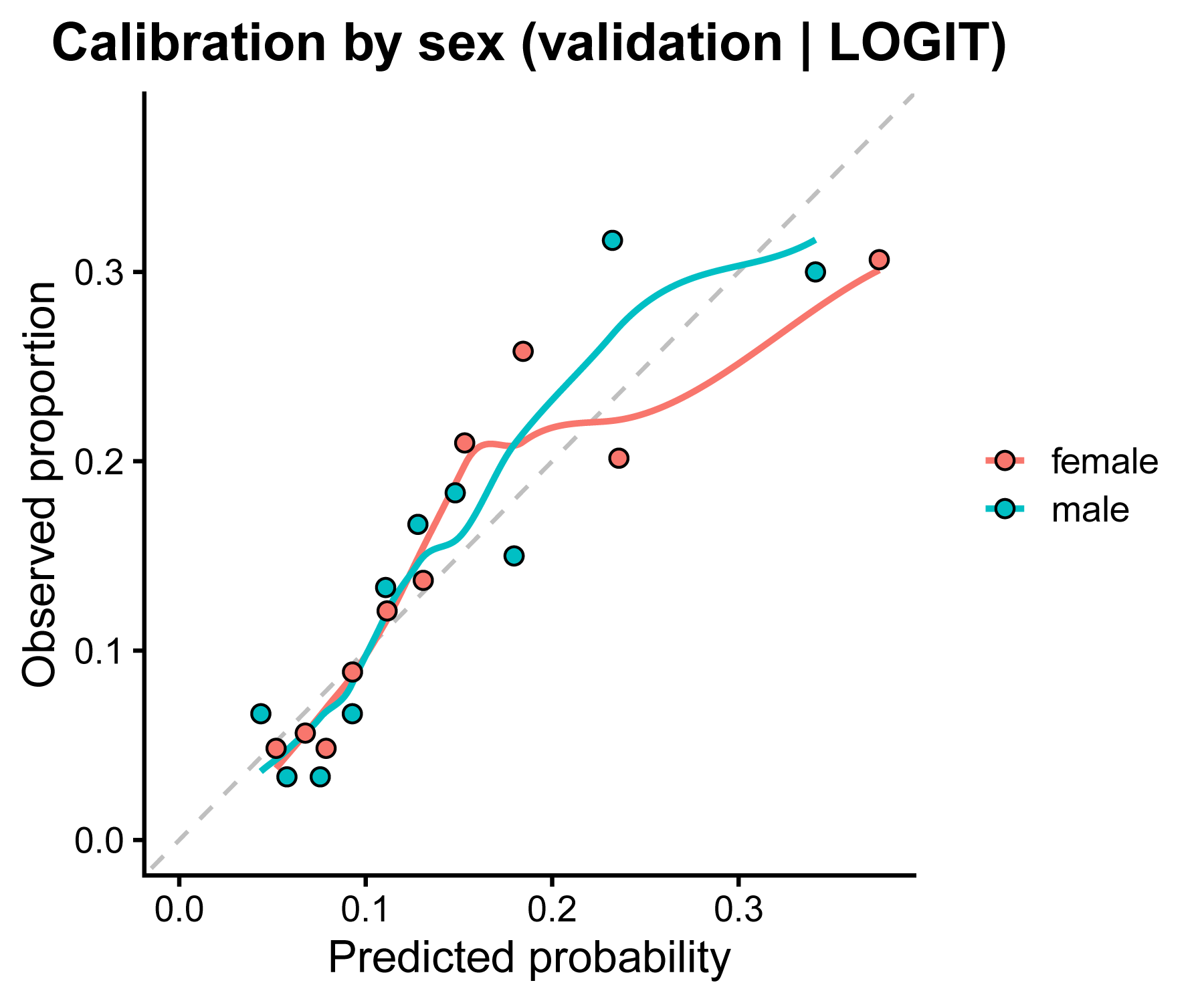

Supplement: Supplementary file 3 [file mmc3.zip › mmc3.tif]

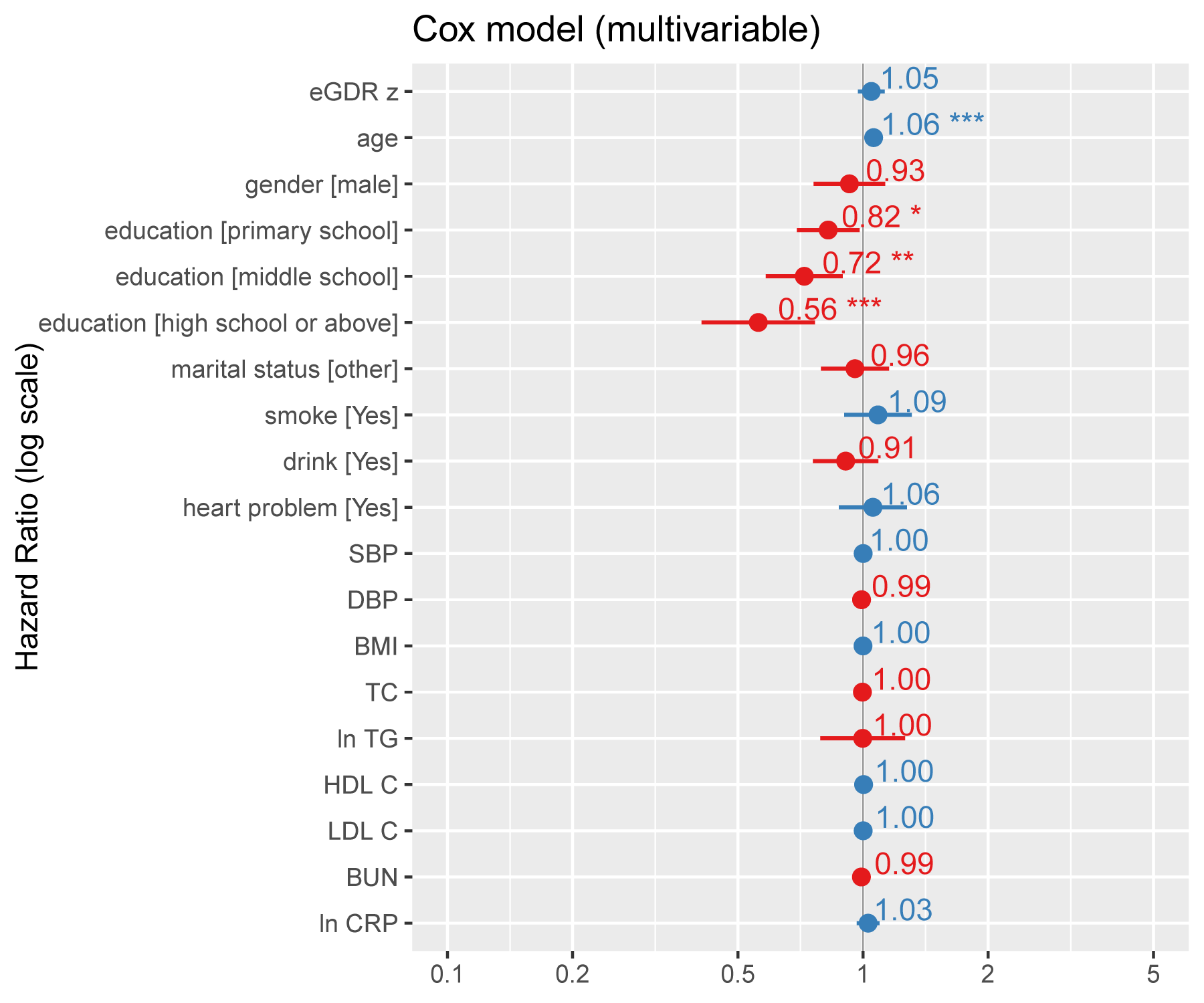

Supplement: Supplementary file 4 [file mmc4.zip › mmc4.tif]

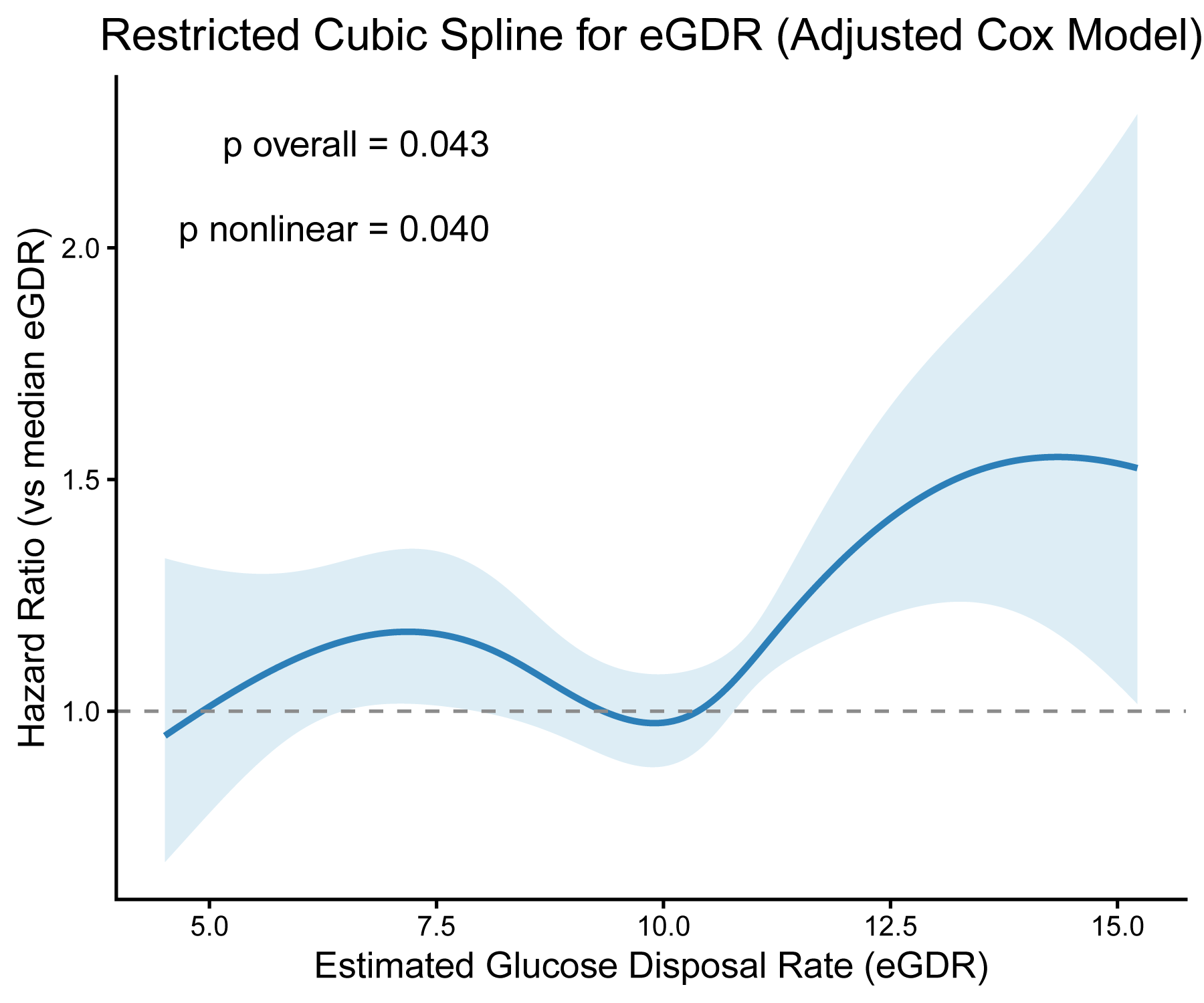

Supplement: Supplementary file 5 [file mmc5.zip › mmc5.tif]
